# Pattern of Primary Resistance of *Helicobacter pylori* to Clarithromycin among Pediatric Patients from North-Eastern Romania

**DOI:** 10.3390/children10111752

**Published:** 2023-10-28

**Authors:** Oana-Maria Rosu, Nicoleta Gimiga, Roxana Popescu, Ileana Ioniuc, Carmen Daniela Rusu, Tatiana Clipa, Diana-Maria Florea, Doina-Anca Pleșca, Alexandru Nemtoi, Elena Tataranu, Gabriela Stefanescu, Smaranda Diaconescu

**Affiliations:** 1 Doctoral School, “George Emil Palade” University of Medicine, Pharmacy, Science, and Technology of Targu Mures, 38 Gheorghe Marinescu Str., 540139 Targu Mures, Romania; 2 Faculty of Medicine, “Grigore T. Popa” University of Medicine and Pharmacy, 16 Universitatii Str., 700115 Iasi, Romania; 3 Medical Genetics Department, “Cuza Voda” Clinical Hospital of Obstetrics and Gynecology, 34 Cuza Voda Str., 700038 Iasi, Romania; 4 Faculty of Medicine, “Carol Davila” University of Medicine and Pharmacy, 21 Dionisie Lupu Str., 020021 Bucharest, Romania; 5 Faculty of Medicine and Biological Sciences, “Stefan cel Mare” University of Suceava, 13 Universitatii Str., 720229 Suceava, Romania; 6 Faculty of Medicine, “Titu Maiorescu” University of Medicine, 67A Gheorghe Petrascu Str., 031592 Bucharest, Romania

**Keywords:** children, *Helicobacter pylori*, antibiotic resistance

## Abstract

Background: *Helicobacter pylori* antibiotic resistance has increased worldwide and affects the effectiveness of current therapies. The recommended first-line empiric treatment should be tailored to the local clarithromycin resistance rate. This study aimed to determine the pediatric patient profile and rate of clarithromycin resistance for patients diagnosed with *Helicobacter pylori* by gastric biopsy. Methods: We studied 84 positive gastric samples for *Helicobacter pylori*. Positive results were confirmed by a rapid urease test and histopathological examination, with the type of gastritis established according to the Sydney System. Gastric biopsy samples were stored in RNA saver. Clarithromycin resistance was determined by a real-time polymerase chain reaction-based molecular assay after RNA-DNA extraction. Results: Of the 84 biopsy samples analyzed, 35 (41.6%) were resistant to clarithromycin. Clarithromycin resistance was found mainly in girls (80%) with a mean age of 15 years (range 6–17 years). The history of prior exposure to clarithromycin was 91.6%. The concordance between the histopathological examination and the PCR test was 100%. Conclusions: One in 2.4 children infected with *Helicobacter pylori* had a strain resistant to clarithromycin. This resistant strain may be a reason for treatment failure in Romanian children, yet this is uninvestigated. The high rate of bacterial resistance to this antibiotic among children indicates the need for susceptibility testing before therapy.

## 1. Introduction

*Helicobacter pylori* (*H. pylori*) infection represents a challenge in medical practice. This infection can be diagnosed at any time during life and is spread globally, especially in developing countries [1,2]. Untreated, *H. pylori* can cause digestive diseases, the most severe being gastric cancer and gastric mucosa-associated lymphoid tissue (MALT) lymphoma [3,4]. The ways of transmission are person-to-person, oral, or oral–fecal [5].

In Romania, the prevalence of the infection reaches a percentage of 68% in adults [3,6] and between 33% and 45% in children [7,8]. *H. pylori* infection in pediatrics occurs most often in the first years of life in developing countries. The prevalence rate in these countries is associated with low socioeconomic status and poor hygiene conditions [3].

The diagnosis of gastric infection with *H. pylori* in children is established according to the updated European Society for Pediatric Gastroenterology, Hepatology, and Nutrition (ESPGHAN), and the North American Society for Pediatric Gastroenterology, Hepatology, and Nutrition (NASPGHAN) guidelines [9]. For pediatric patients, the gold standard in diagnosis is upper digestive endoscopy with gastric biopsy sampling. A positive bacterial culture or histopathology (*H. pylori*-positive gastritis) plus at least one other positive biopsy-based test is required. Gastric biopsy-based tests include the rapid urease test (RUT), or molecular tests if available, including fluorescent in situ hybridization or polymerase chain reaction (PCR) [9]. Histopathological changes detected on gastric biopsy samples should be systematized according to the updated Sydney classification for gastritis [10]. Eradication antibiotic therapy is limited in the case of pediatric patients, the recommended antibiotics being clarithromycin (CLR), metronidazole (MTZ), and amoxicillin (AMX).

According to current ESPGHAN/NASPGHAN guidelines, treatment to eradicate *H. pylori* gastric infection in children should include two antibiotics (amoxicillin, clarithromycin, or metronidazole) and a proton pump inhibitor (PPI) [9]. Unfortunately, acquired antibiotic resistance is often encountered and is an important cause of treatment failure. Various rates of bacterial resistance to antibiotics used in therapeutic regimens have been reported, especially for clarithromycin [11].

Rates of resistance to primary antibiotics are high, with large differences between geographic regions [12]. In Europe, resistance to CLR is 28% in the northern region, and in southern countries, the percentage is 7% [13]. Current treatment guidelines recommend not using CLR when resistance rates exceed >15% [14]. The World Health Organization (WHO) in 2017 classified *H. pylori* as a pathogen with high priority in the research and development of new antibiotics due to increased resistance to CLR [15].

This study aims to describe *H. pylori* infection in children by determining the pediatric patient profile, symptomatology and endoscopic appearance. We also gathered information on the rate of CLR resistance in a cohort of pediatric patients based on the PCR testing of gastric biopsies and correlation with the Sydney Gastritis Classification System. 

## 2. Materials and Methods

We conducted a prospective study in the Pediatric Gastroenterology Clinic of the St. Mary Children’s Hospital, Iasi, Romania. The research was carried out over a period of 20 months and had the approval of the Ethics and Research Committee of the hospital, registered with no. 4363/20.02.2019.

The research enrolled patients aged 6 to 17 years who underwent upper gastrointestinal endoscopy for symptoms or complaints: epigastric pain, recurrent abdominal pain, or vomiting. A positive result of *H. pylori* infection on histopathological examination and rapid urease test was required. Informed consent was signed by the parents or guardians of the children included in the study under the protection of anonymity. After discharge from the hospital, a questionnaire was completed via phone and personal data were protected to preserve anonymity. The purpose of the questionnaire was to collect information about the pediatric patient such as the area of residence or antibiotic history.

Inclusion criteria: children between 6 and 18 years of age, *H. pylori* infection confirmed by rapid urease test and histopathology, and consent to participate in the study. Exclusion criteria: age outside the range of 6–17 years, patients with associated pathologies (such as celiac disease or Crohn’s disease), and patients who had a history of *H. pylori* infection.

Gastric biopsy samples were stored in RNA-save solution and later processed due to the COVID-19 pandemic. Due to the pandemic overlapping with our study, we did not have access to the RT-PCR device. Patients were treated according to current recommendations to receive eradication treatment therapy based on unknown resistance with high-dose PPI-AMO-MET for 14 days.

Gastric biopsy samples were stored in RNA-save (Biological Industries Israel Beit Haemek Ltd., Haemek, Israel). This solution preserves and allows the recovery of intact RNA from tissues. ARN Save should be stored at room temperature. Samples stored in solution can be stored indefinitely at −20 °C or −80 °C without RNA degradation. The gastric biopsy samples from the RNA-save solution were stored in sterile Expell 2.0 mL graduated microcentrifuge tubes (CAPP, Nordhause, Germany).

Viasure Real-Time PCR Detection kit (by CerTest, Biotec, Zaragoza, Spain) was used to determine the resistance of the bacteria to antibiotics. This kit provides specific identification of *H. pylori* and detects CLR resistance from gastric biopsies in patients presenting with gastrointestinal signs and symptoms. DNA was extracted from the biopsy sample, multiplied using real-time amplification, and detected using specific primers and a fluorescent reporter dye probe for *H. pylori* and CLR. In order to determine the correctness of the method, 30 gastric biopsy samples negative to other methods were processed in order to determine the absence of *H. pylori* using this method as well.

Continuous variables are presented as mean ± standard deviation and are compared using Student’s *t* test. Categorical variables are expressed as numbers and/or percentages and are compared with the Chi-square test. Pearson correlations were used for quantitative data analysis. All statistical tests are two-tailed and *p* < 0.05 was considered statistically significant. All statistical analyses were performed in IBM SPSS Statistics (Statistical Package for the Social Sciences) for Windows, version 20 (Armonk, NY, USA).

## 3. Results

Eighty-four patients who corresponded to the inclusion and exclusion criteria were eligible for this research. Among the patients enrolled in the study, 60 (71.5%) are girls, and 24 (28.5%) are boys. Thirty come from urban areas (35.7%), and 54 from rural areas (64.3%). We divided these patients into three age ranges: 6–9 years (10 patients, 11.9%), 10–14 years (32 patients, 38.1%), and 15–17 years (42 patients, 50%). 

The resistance of *H. pylori* to CLR was determined as follows: 35 patients have resistant *H. pylori* (41.6%), and 49 have non-resistant *H. pylori* (58.4%). The data of patients participating in the research were stored and centralized to create the database (Table 1).

To differentiate the characteristics of patients with *H. pylori* infection, we divided them into two groups: CLR-resistant and CLR-non-resistant. A comparative study of patients with *H. pylori* infection is detailed in Table 2. 

In both groups, resistant to CLR and non-resistant to CLR, the percentage of girls affected by *H. pylori* infection is higher than that of boys (80.0% and 65.3%). In both groups, the infection is more frequent with increasing age. The most common addressable symptom was epigastric pain, followed by vomiting. 

Table 3 lists the 49 patients with *H. pylori* non-resistant to CLR. Of these, 32 (65.3%) are girls, and 17 (34.7%) are boys. The registered age categories are seven patients aged between 6 and 9 years (14.3%), 17 patients aged between 10 and 14 years (34.7%), and 25 patients aged between 15 and 17 years (51.0%). Regarding the place of origin, 13 (37.1%) come from the urban area and 22 (62.9%) come from the rural area. According to living conditions, we found that 12 (34.3%) live in overcrowded housing. Among them, 43 patients (87.8%) had a history of antibiotic therapy with CLR. The symptoms that determined the performance of upper digestive endoscopy were forty-two patients with epigastric pain (85.7%), four patients with recurrent abdominal pain (8.2%), twelve patients with vomiting (24.5%), one patient with treatment-refractory anemia (2.0%), and other symptoms for five patients (10.2%). The endoscopic pattern recorded was the nodular appearance in 31 of the cases (63.3%), erosive appearance in 6 cases (12.2%), hyperemic appearance in 15 patients (30.6%), and snakeskin pattern in 18 patients (36.7%). 

In Table 3, we also present the 35 patients with *H. pylori* resistant to CLR. Of these, 28 are girls, and 7 are boys. The registered age categories are three patients aged between 6 and 9 years (8.6%), 15 patients aged between 10 and 14 years (42.8%), and 17 patients aged between 15 and 17 years (48.6%). Regarding the area of origin, 13 come from the urban environment and 22 come from the urban environment. According to living conditions, we found that 12 (34.3%) live in overcrowded housing. Thirty-four patients (97.1%) had a history of antibiotic therapy with CLR. The symptoms that determined the performance of upper digestive endoscopy were twenty-five patients with epigastric pain, eight patients with recurrent abdominal pain, ten patients with vomiting, three patients with anemia refractory to treatment, and other symptoms for two patients. The endoscopic pattern recorded was nodular appearance in 21 of the cases (60%), erosive appearance in 5 cases (14.3%), hyperemic appearance in 11 patients (31.4%), and snakeskin pattern in 15 patients (42.9%). 

For all the patients included in the study, regardless of resistant or non-resistant *H. pylori*, we tried to establish the type of gastritis according to the Sydney System (Table 4). After analyzing the gastric biopsy obtained by upper digestive endoscopy, according to the staging, neither gastric atrophy nor intestinal metaplasia was determined. A higher density of *H. pylori* is observed among patients with mild gastritis and moderate gastritis, presenting a statistically significant association.

Table 5 reports the *p*-values of the estimated correlations between the Sydney System and age, sex, history of previous antibiotic use, or endoscopic appearance. *H. pylori* density correlates with gender for mild and moderate gastritis. Also, the density of the bacteria correlates with the previous history of antibiotic therapy with clarithromycin and with the nodular appearance in severe gastritis. 

## 4. Discussion

*H. pylori* infection can be challenging when it needs to be diagnosed in pediatric patients. In adults, the diagnosis of gastric infection with *H. pylori* can be based on invasive tests for the gastric biopsy sample taken during upper digestive endoscopy (such as rapid urease test, histology, and culture) or by non-invasive methods (such as serological tests–antibody tests, urea breath test, or fecal antigen) [16,17]. However, for pediatric patients, according to the current ESPGHAN/NASPGHAN guidelines, the diagnosis of *H. pylori* infection in children must be based on positive culture or histopathology plus at least one other positive biopsy-based test, such as the rapid urease test or PCR [9]. 

It is well known that some *H. pylori* detection methods can present a low sensitivity and can determine a false-negative result [18]. On the other hand, the urea breath test can determine positive results more than the reference method [19]. In the study by Jeon et al., the results obtained by PCR vs. RUT were concordant, thus suggesting that PCR testing is an efficient method of detecting *H. pylori* [20]. Bogiel et al. conducted a study on children where they saw a high concordance between the results of *H. pylori* detection using histopathological investigation and molecular methods [21]. Also, in the study conducted by Srebinska et al., most cases were correctly diagnosed by the real-time PCR method [22]. Some authors claim that UBT and histology, like classical methods, have similar accuracy and could be verified by the PCR method only in some instances [23,24]. Similar to other research, our study applied the real-time PCR method. This method also determines the presence or absence of *H. pylori* in gastric biopsy samples. The real-time PCR method quickly and correctly identified the presence of bacteria in biopsy samples. 

Our research shows that *H. pylori* infection in pediatric patients can be statistically significantly correlated with epigastric pain (*p* = 0.048), contrary to other studies. Spee et al. demonstrate in their study that there is no correlation between the symptoms described by the patients and *H. pylori* infection [25]. As in the survey conducted by Helmbold et al., no significant correlations between symptoms and illness were demonstrated [26]. 

Our study revealed a statistically significant association between the endoscopic appearance of nodularity and the increased density of *H. pylori* in the Sydney System (*p* = 0.009). Several studies have reported correlations between endoscopic appearance and the histological diagnosis of gastritis, especially in children [27,28]. Some studies claim that the nodular appearance at upper digestive endoscopy predicts *H. pylori* infection for pediatric patients [29,30]. In the survey carried out by Ozbey et al., antral nodularity was detected in 54.5% of the children, and 76.4% of them had a positive result for *H. pylori* [31]. Several studies with variable percentages of endoscopic nodularity in pediatric patients support the correlation between *H. pylori* infection and the endoscopic appearance of nodular gastritis [32]. Kalach et al. performed a meta-analysis on the histopathological aspects of gastric biopsy according to the Sydney System in children, which revealed that there was a higher risk of chronic inflammation, and atrophy occurred rarely [33]. Similar to this study, our research reveals that all patients present various degrees of chronic inflammation and no atrophic aspect was recorded. 

According to the Sydney System, the histopathological diagnosis of gastritis does not correlate statistically significantly with gender (*p* > 0.05). Still, there is a statistically significant correlation with age for the forms that present *H. pylori* in medium (*p* = 0.021) and light density (*p* = 0.006). Similarly, in the study by Urganci et al., patients with antral nodularity showed gastritis with *H. pylori*, which is significantly associated with older age and the histological density of the bacteria [34]. Numerous studies report significant correlations between the nodular endoscopic appearance and the density of *H. pylori* colonization [35,36], but there are also some contradictory studies [37]. Koh et al. report a significant increase in the incidence of nodular gastritis but without a statistically significant association with gender or age [38]. Urganci et al. detect a significant association between the increase in endoscopic nodularity with the increase in *H. pylori* density [34].

Our study reveals a statistically significant association between a history of antibiotic therapy and a histopathological diagnosis of gastritis. This association was determined between previous records of CLR therapy and *H. pylori* density (*p* = 0.001). Gazi et al. compared histopathological data with bacterial resistance and observed a significant correlation between histological aspects of gastritis (such as inflammation severity and bacterial density) and PCR-determined CLR resistance [39].

Eradication therapy can be successful depending on the bacterial resistance in a particular region and the population under investigation. A treatment performed and failed can increase the risk of resistance to *H. pylori* [40]. The primary resistance to CLR increased above the 15% level recommended by the ESPGHAN/NASPGHAN guidelines, with it being necessary to determine the regional antibiotic resistance to make the eradication scheme more efficient [41].

In children, global studies report an increased incidence of antibiotic-resistant primary strains, such as 55.2% for CLR and 71.3% for MTZ [42,43]. Amoxicillin is usually used in first-line eradication therapy. The effectiveness of this antibiotic has been investigated through studies in adults and children, and its resistance remains low [44].

According to the statistical analysis performed in our study, no statistically significant correlation was detected between H. pylori resistance to antibiotics and age, gender, or environment (*p* > 0.05). However, there is an increased number of infected patients with age. For patients with *H. pylori* resistant to CLR, the percentages per age group are 6–9 years with 8.6%, 10–14 years with 42.8%, and 15–17 years with 48.6%. The finding may suggest that with increasing age, exposure to various sources of infection also increases, which may explain the higher infection rate among school-age children. Moreover, the infection rate can be higher in children attending schools with unsanitary conditions [45]. This pattern of the increasing prevalence of *H. pylori* infection in children with age is similar to what other research has shown [46,47,48].

In our study, a statistically significant association was detected between the resistance of the bacteria and the history of antibiotic therapy with CLR (*p* = 0.046). In the research carried out by Burayzat et al., strains isolated from patients who had previously received CLR were determined to be resistant to CLR in a proportion of 70% [49]. Also, the study by Le et al. claims that the frequency of CLR resistance has increased significantly, especially in patients who previously received treatment with this antibiotic [50].

In our study, CLR resistance was statistically significantly correlated with the Sydney System when there was severe *H. pylori* density (*p* = 0.001). Some studies demonstrate that gastric biopsies from pediatric patients generally have significant colonization with reduced inflammation [51] and a greater gastric inflammatory response than the density of the infection itself [52,53].

Our study has some limitations. The research was conducted in a single hospital and did not target the general population. Also, the treatment and eradication rate are separate from this article. No data on the trade name or preparation used from the previous antibiotic regimens of the patients enrolled in the study were recorded. The study has a small sample size, which determines that the observed data do not provide strong enough evidence to reject the null hypothesis, but do provide a database for further investigation of the subject, having a precise importance in pediatric medical practice. In addition, our study presents recent data from current clinical practice and contains valuable information on bacterial resistance, as studies in children are limited. 

Antibiotic resistance rates are not constant and may differ between regions. Multicenter studies are needed in order to establish the local resistance rate to clarithromycin in order to make the eradication of *H. pylori* infection more effective in Romania.

## 5. Conclusions

This research provides evidence of what the profile of the patient infected with clarithromycin-resistant *H. pylori* might look like. An important factor is female sex, rural origin, advanced age, and, last but not least, the history of exposure to clarithromycin.

Also, the study provides important information regarding the resistance of *H. pylori* to clarithromycin among Romanian children. Research determines a rate of resistance to clarithromycin of 41.6%. Extensive studies are needed in Romania at several medical centers to determine the regional resistance rate. The eradication treatment commonly used by pediatricians and family doctors would need to be chosen according to a resistance greater than 15% or according to an unknown resistance.

These preliminary data could determine changes in *H. pylori* eradication schemes in the future. In most medical centers, there is no possibility of antibiotic resistance testing. These tests should at least be performed in hospitals with a pediatric gastroenterology department. Performing a single RT-PCR test to identify *H. pylori* and to determine microbial resistance is a valuable method for treating the infection.

Therefore, we believe that regional profiling of clarithromycin resistance is essential to reduce the risk of failure of eradication therapy.

## Figures and Tables

**Table 1:** Study population characteristics.

Characteristics	Variables	Number of Patients	%
Sex	Female	60	71.5%
	Male	24	28.5%
Age	6–9 years	10	11.9%
	10–14 years	32	38.1%
	15–17 years	42	50%
Place of residence	Urban	30	35.7%
	Rural	54	64.3%
*H. pylori* * resistance	Resistance	35	41.6%
	Non-resistance	49	58.4%

^*^ Helicobacter pylori.

**Table 2:** The comparative characteristics of the studied patients.

Characteristics		Resistant to CLR *		Non-Resistant to CLR *		*p*
		n	%	n	%	
Sex	Female	28	80.0	32	65.3	0.145
	Male	7	20.0	17	34.7	
Age	6–9 years	3	8.6	7	14.3	0.868
	10–14 years	15	42.9	17	34.7	
	15–17 years	17	48.6	25	51.0	
Clinical symptoms	Epigastric pain	25	71.4	42	85.7	0.819
	Abdominal recurrent pain	8	22.9	4	8.2	
	Vomiting	10	28.6	12	24.5	
	Refractory anemia	3	8.6	1	2.0	
	Other	2	5.7	5	10.2	

* CLR–clarithromycin;

**Table 3:** Characteristics of patients with *H. pylori* strains.

Characteristics		Non-Resistant to CLR		Resistant to CLR		*p*
		*n*	%	*n*	%	
Sex	Female	32	65.3	28	80.0	0.063
	Male	17	34.7	7	20.0	
Age	6–9 years	7	14.3	3	8.6	0.058
	10–14 years	17	34.7	15	42.9	
	15–17 years	25	51.0	17	48.6	
Environment	Urban	17	34.7	13	37.1	0.082
	Rural	32	65.3	22	62.9	
Living conditions	1 pers/room	16	32.6	6	17.1	0.075
	2 pers/room	22	44.9	17	48.6	
	≥3 pers/room	11	22.5	12	34.3	
History of antibiotic therapy	CLR	43	87.8	34	97.1	0.046
	MTZ	8	16.3	6	17.1	
	AMX	7	14.3	4	11.4	
	AMC	27	55.1	19	54.3	
	Other	8	16.3	12	34.3	
	Cannot remember	1	2.0	0	0.0	
Clinical symptoms	Epigastric pain	42	85.7	25	71.4	0.048
	Abdominal recurrent pain	4	8.2	8	22.9	
	Vomiting	12	24.5	10	28.6	
	Refractory anemia	1	2.0	3	8.6	
	Other	5	10.2	2	5.7	
Endoscopic pattern	Nodular	31	63.3	21	60.0	0.078
	Erosive	6	12.2	5	14.3	
	Hyperemic	15	30.6	11	31.4	
	Snakeskin	18	36.7	15	42.9	

CLR—clarithromycin; MTZ—metronidazole; AMX—amoxicillin; AMC—amoxicillin/clavulanic acid.

**Table 4:** Histopathological profile according to the Sydney System correlated with *H. pylori* infection.

Characteristics	None		Mild		Moderate		Severe		*p*
	n	%	n	%	n	%	n	%	
Chronic Inflammation	-	-	17	20.2	56	66.6	11	13.1	0.072
Neutrophilic Infiltrate	15	17.9	28	33.3	34	40.5	7	8.3	0.062
Atrophy	84	100	-	-	-	-	-	-	-
Intestinal Metaplasia	84	100	-	-	-	-	-	-	-
*H. pylori* Density	1	1.2	44	52.4	27	32.1	12	14.3	0.045 *

* Significant *p* value < 0.05.

**Table 5:** Correlations between the Sydney System and age, sex, history of antibiotic therapy, and endoscopic pattern.

Sydney System			Age	Sex	History of Antibiotic Therapy				Endoscopic Pattern			
					CLR	MTZ	AMX	AMC	Nodular	Erosive	Hyper Emic	Snake Skin
Chronic Inflammation	None	CC	−0.069	0.086	0.033	−0.049	−0.045	−0.133	−0.140	−0.043	0.164	−0.088
		*p*-value	0.53	0.44	0.76	0.66	0.69	0.23	0.20	0.70	0.14	0.42
	Mild	CC	0.096	−0.203	0.037	0.027	0.062	−0.094	0.006	−0.009	0.069	−0.142
		*p*-value	0.39	0.06	0.74	0.81	0.58	0.39	0.96	0.94	0.53	0.20
	Moderate	CC	−0.056	0.196	−0.030	−0.158	0.000	0.034	0.017	0.050	−0.073	0.155
		*p*-value	0.61	0.07	0.78	0.15	1.00	0.76	0.88	0.65	0.51	0.16
	Severe	CC	−0.011	−0.065	−0.011	0.205	−0.058	0.104	0.014	−0.046	−0.031	−0.023
		*p*-value	0.92	0.56	0.92	0.06	0.60	0.34	0.90	0.68	0.78	0.83
Neutrophilic Infiltrate	None	CC	−0.069	0.012	0.033	−0.049	−0.045	0.091	0.086	−0.043	−0.073	−0.088
		*p*-value	0.53	0.91	0.76	0.66	0.67	0.41	0.44	0.70	0.51	0.42
	Mild	CC	0.073	0.083	0.023	0.034	−0.062	−0.056	0.015	0.111	−0.130	0.125
		*p*-value	0.51	0.45	0.83	0.76	0.57	0.61	0.89	0.32	0.24	0.26
	Moderate	CC	−0.38	−0.001	0.073	−0.174	0.079	0.136	0.098	−0.104	0.025	−0.067
		*p*-value	0.73	0.99	0.51	0.11	0.47	0.21	0.38	0.34	0.82	0.54
	Severe	CC	0.00	−0.171	−0.065	0.212	0.000	−0.015	−0.030	−0.117	0.078	−0.066
		*p*-value	1.0	0.12	0.56	0.05	1.00	0.89	0.79	0.29	0.48	0.55
H. pylori Density	None		-	-	-	-	-	-	-	-	-	-
	Mild	CC	−0.015	0.296	0.050	0.053	−0.078	0.117	0.019	−0.115	−0.119	0.054
		*p*-value	0.89	0.01 *	0.65	0.63	0.48	0.29	0.87	0.30	0.28	0.63
	Moderate	CC	0.185	−0.252	−0.069	0.034	0.083	−0.108	−0.142	0.035	0.091	−0.032
		*p*-value	0.09 *	0.02 *	0.53	0.76	0.45	0.33	0.20	0.75	0.41	0.77
	Severe	CC	−0.198	−0.087	0.129	−0.103	0.013	−0.049	0.132	0.029	0.070	−0.075
		*p*-value	0.07	0.43	0.001 *	0.66	0.69	0.41	0.009 *	0.79	0.53	0.50

CC–Correlation coefficient; (*)-significant *p* value (*p <* 0.05).

## Data Availability

Not applicable.
